# A Previously Discounted Flap Now Reconsidered: MatriDerm and Split-Thickness Skin Grafting for Tendon Cover Following Dorsalis Pedis Fasciocutaneous Flap in Lower Limb Trauma

**Published:** 2014-04-28

**Authors:** Jonathan A. Dunne, Daniel J. Wilks, Jeremy M. Rawlins

**Affiliations:** ^a^Department of Plastic Surgery, St George's Hospital, Blackshaw Road, London, SW17 0QT, UK; ^b^Department of Plastic Surgery, Leeds General Infirmary, Great George Street, Leeds, LS1 3EX, UK; ^c^Department of Plastic Surgery and Burns, Royal Perth Hospital, Wellington Street, Perth 6000, Australia

**Keywords:** dorsalis pedis flap, reconstruction, MatriDerm, skin, lower limb trauma

## Abstract

**Objective:** The dorsalis pedis flap has reliable vascularity; however, its use is limited by reports of donor site morbidity including infection, delayed healing, exposure of tendons, and later contractures. The purpose of this study was to demonstrate its continued role in lower limb trauma when the donor site is reconstructed with MatriDerm to avoid complications. **Methods:** A 65-year-old man presented with a displaced, Gustilo 3b open transverse fracture of his left distal fibula. He had a 2 cm^2^ open wound over his lateral malleolus. **Results:** Following review of possible local options, a dorsalis pedis fasciocutaneous flap was deemed best for coverage, and the donor site was closed with 1-mm MatriDerm dermal matrix and a 6/1000 inch split-thickness skin graft (STSG) in a single stage. Three months postoperatively, the foot had excellent function and cosmesis, with toes in a neutral position and a full range of movement. **Conclusions:** The dorsalis pedis flap is a valuable reconstructive option for defects of the foot and ankle. Its major limitation donor site morbidity can be overcome by the additional application of a dermal substitute such as MatriDerm under the STSG.

## INTRODUCTION

The dorsalis pedis flap was first described by McGraw et al^1^ in 1975 as a local pedicled flap. The flap has a role in local reconstruction of the foot and ankle, or it may be employed as a free tissue transfer where thin, pliable skin is required. It can be used to reconstruct areas such as the hands, where tendons may also be transferred. Advocates cite its reliable vascularity and relatively thin skin, while the depth of the dermis in this flap ensures it is an excellent option for local reconstruction of the foot and ankle.^2^

However, use of the flap is limited by reports of donor site morbidity, with both short- and long-term complications. Soft tissue infections, wound breakdown, and tendon exposure have been reported as early complications, while long-term morbidity may include delayed healing and contractures.^2^ We propose that the application of a dermal substitute and split-thickness skin graft (STSG) to the donor site greatly reduces donor site morbidity and extends the scope of this versatile flap.

## METHODS/CASE PRESENTATION

A 65-year-old man presented acutely having fallen from a skip. He sustained a displaced, Gustilo 3b open transverse fracture of his left distal fibula. He had a 2-cm^2^ open wound over his lateral malleolus. In addition, he had a displaced transverse fracture of the right distal fibula, compression fracture of T12 and L1, and an oblique left calcaneal fracture. He was medicated with bendroflumethiazide and enalapril for hypertension and simvastatin for hypercholesterolemia.

The following day, he underwent manipulation of both fibulae under anesthesia and debridement of soft tissue wounds. One week later, further wound debridement led to exposure of the lateral malleolus. Given the significant comorbidities and time since injury, a free flap was considered too high risk and a local reconstructive option was selected.

Local options were considered, however, the peroneus brevis and reverse sural artery flaps do not provide reliable coverage to the lateral malleolus. A peroneus brevis flap would not have covered this defect (and was in the zone of trauma), while the reverse sural artery flap is not a reliable choice for a patient with polytrauma. The large donor site requiring STSG is very often complicated by the need for a plaster of Paris splint in the postoperative period.

## RESULTS

**Figure 1:**
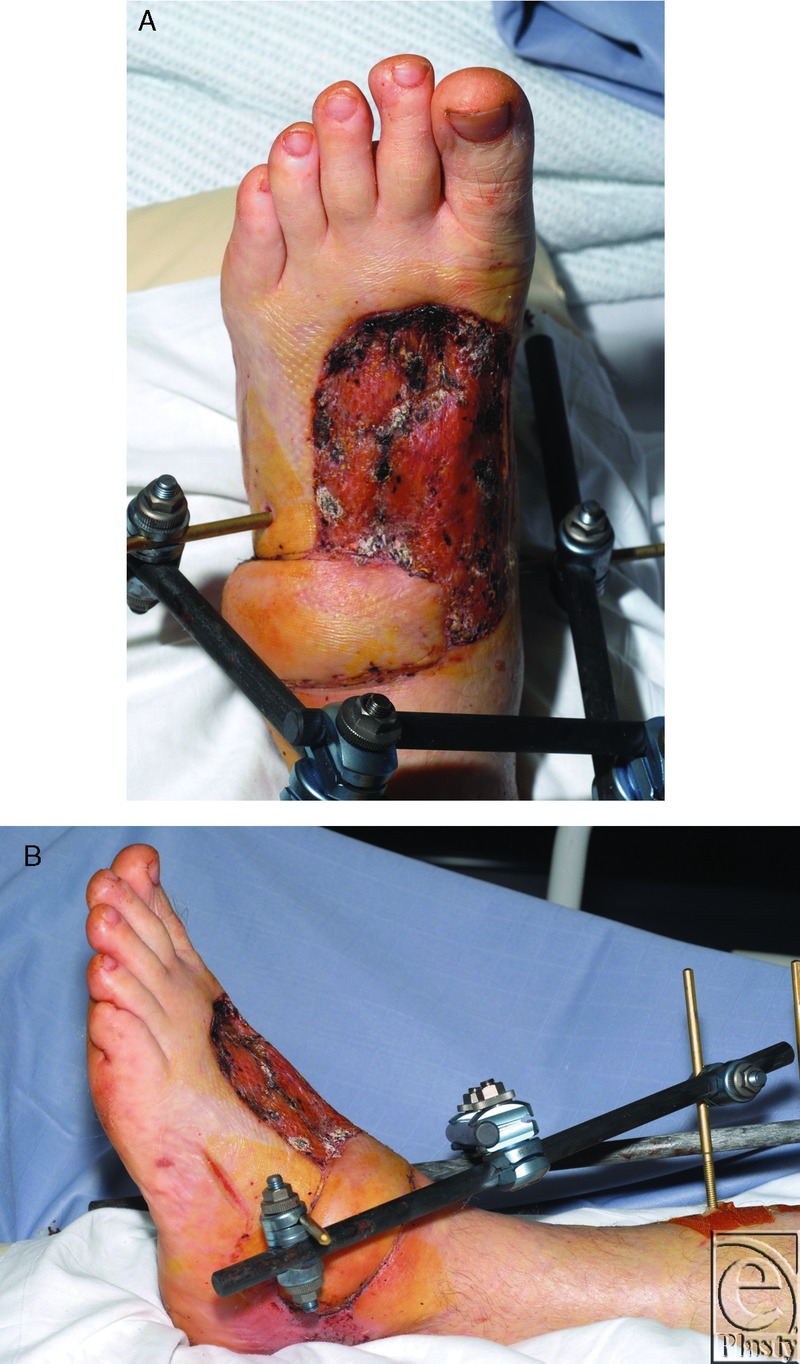
At 10 days post-operatively, dorsum of foot (A) and lateral view (B).

**Figure 2:**
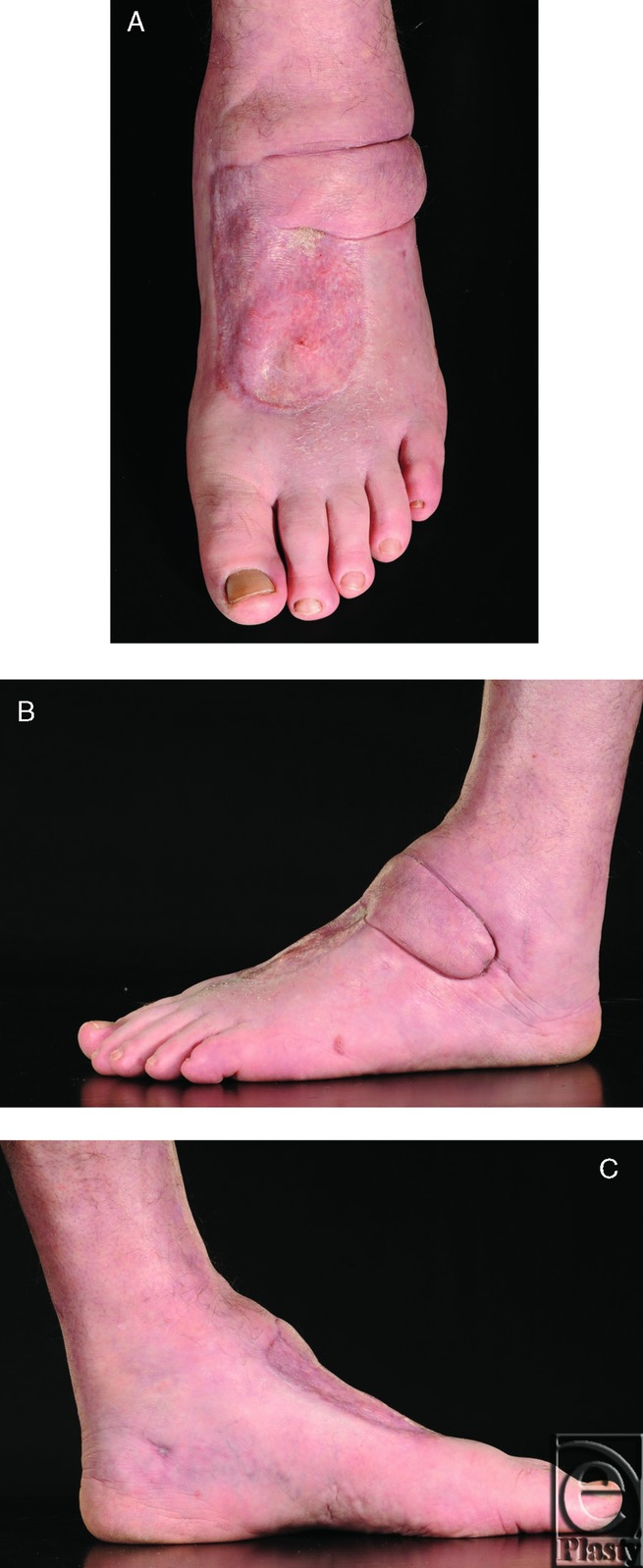
At 3 months post-operatively, dorsum of foot (A), lateral view (B) and medial view (C).

On day 15, after a period of negative pressure therapy, he underwent soft tissue reconstruction using a pedicled dorsalis pedis flap. The flap was elevated in the standard fashion,^1^ transposed, and inset with 4/0 Prolene. One-millimeter MatriDerm (MedSkin Solutions Dr Suwelack AG, Billerbeck, Germany) was positioned over the donor site and a 6/1000-inch STSG was applied over the MatriDerm using Histoacryl glue. The site was dressed with proflavine wool, Mepitel (Mölnlycke Health Care, Gothenburg, Sweden), and wool and crepe. The dressings were left in situ until graft check at day 8 when complete take was observed (Figs 1a and 1b). The flap remained viable throughout and did not require revisional surgery. At 3 months, the donor site had achieved an excellent cosmetic result with a smooth contoured appearance (Figs 2a-2c). His toes assumed a neutral position and displayed a full range of motion.

## DISCUSSION

A local pedicled dorsalis pedis flap is advantageous over free tissue transfer because it provides a good match of skin color and dermal depth, is a reliable flap, and can be performed rapidly. Its limitations are related to morbidity of its donor site; however, using a dermal substitute such as MatriDerm may obviate these challenges.

MatriDerm is a collagen-elastin matrix used to reconstruct the dermis. The dorsalis pedis flap donor site presents challenges of managing a defect with exposed paratenon and significant contours. Split-thickness skin graft (STSG) is most commonly used to cover the defect; however, using such reconstruction alone may be complicated by incomplete graft take and exposed tendons, tendon tethering, and contractures of the toes. Haslik et al^3^ examined use of MatriDerm in severe burns to the hands, in conjunction with unmeshed skin grafting. At 3 months, there was excellent pliability of the wounds with a full range of movement of the hand. The use of MatriDerm in conjunction with STSG in burns surgery improves skin elasticity compared to STSG alone,^4^ which may reduce contractures in the longer term.

MatriDerm has been reported principally for upper limb defects, in particular the hand. Similar to the dorsalis pedis flap donor site, such areas are prone to stress and have limited skin coverage over tendons and joints. Full-thickness defects in the distal upper limb demonstrate excellent revascularization in a single-stage reconstruction with MatriDerm and STSG.^5^ Longer-term follow-up demonstrates excellent scar appearance and suggests this procedure offers a long-term solution.

The dorsalis pedis flap is a valuable reconstructive option for defects of the foot and ankle. Its major limitation, donor site morbidity, can be overcome by the additional application of a dermal substitute such as MatriDerm under the STSG.
